# Approaches towards the synthesis of a novel class of 2-amino-5-arylazonicotinate, pyridazinone and pyrido[2,3-*d*]pyrimidine derivatives as potent antimicrobial agents

**DOI:** 10.1186/1752-153X-7-123

**Published:** 2013-07-17

**Authors:** Hamada Mohamed Ibrahim, Haider Behbehani, Mohamed H Elnagdi

**Affiliations:** 1 Chemistry Department, Faculty of Science, Kuwait University, P.O. Box 5969, Safat 13060, Kuwait; 2 Chemistry Department, Faculty of Science, Fayoum University, Fayoum 63514, A. R., Egypt

**Keywords:** Azonicotinates, Pyridazinones, Pyrido[2,3-*d*]pyrimidine, X-ray, Antimicrobial activity

## Abstract

**Background:**

Despite significant progresses in antimicrobial therapy, infectious diseases caused by bacteria and fungi remain a major worldwide health problem because of the rapid development of resistance to existing antimicrobial drugs. Therefore, there is a constant need for new antimicrobial agents. There are a large number of heterocyclic derivatives containing nitrogen atoms that possess a broad spectrum of biological activities including pyridine and pyridazine, which are two of the most important heterocycles in medicinal chemistry.

**Results:**

The reaction of 3-oxo-2-arylhydrazonopropanals **2** with ethyl cyanoacetate and malononitrile **3a,b** has led to the formation of 2-amino-5-arylazo-6-aryl substituted nicotinates **8a-k** as sole isolable products when the aryl group in the arylazo moiety was substituted with an electron-withdrawing group like Cl, Br, NO_2_. The pyridazinones **10** were formed from the same reaction when the arylazo moiety was phenyl or phenyl substituted with an electron-donating group. The 2-aminoazonicotinates **8** were condensed with DMF-DMA to afford the amidines **13a,b**, which then were cyclized to afford the targeted pyrido[2,3-*d*]pyrimidine derivatives **15a,b,** respectively. The structures of all new substances prepared in this investigation were determined by using X-ray crystallographic analysis and spectroscopic methods. Most of the synthesized compounds were tested and evaluated as antimicrobial agents and the results indicated that many of the obtained compounds exhibited high antimicrobial activity comparable to ampicillin, which was used as the reference compound.

**Conclusion:**

A general rule for the synthesis of 2-amino-5-arylazo-6-aryl substituted nicotinic acid and pyridazinone was established using 3-oxo-2-arylhydrazonopropanal as a precursor. Moreover, a novel route to pyrido[2,3-*d*]pyrimidine was achieved. Most of the synthesized compounds were found to exhibit strong inhibitory effects on the growth of Gram-positive bacteria especially *Bacillus subtilis*. Compounds **1a, 8a-h**, **10a-c**, **15b** and **16** showed a broad spectrum of antimicrobial activity against *B. subtilis*.

## Background

The emergence and spread of antimicrobial resistance has become one of the most serious public health concerns across the world. Antimicrobial resistance refers to microorganisms that have developed the ability to inactivate, exclude, or block the inhibitory or lethal effects of antimicrobial agents [1]. Despite significant progress in antimicrobial therapy, infectious diseases caused by bacteria and fungi remain a major worldwide health problem because of the rapid development of resistance to the existing antimicrobial drugs (antibacterial and antifungal). In other words, the increasing use and misuse of existing antimicrobial drugs have resulted in the development of resistant pathogens. In particular, the emergence of multidrug-resistant Gram-positive and -negative bacteria has caused life-threatening infectious diseases in many countries. The chemical and biological study of heterocyclic compounds has been of interest for many years for medicinal and agricultural reasons. There are a large number of heterocyclic derivatives containing nitrogen atoms such as pyridine and pyridazine that possess a broad spectrum of biological activities including antimicrobial [2-6], anti-inflammatory and analgesic [7-9], anti-HIV [10], antiplasmodial [11], anti-tubercular [3,12], antibacterial [3,13], anticonvulsant [14,15], inhibition of cyclo-oxygenase [16], antidiabetic [17], antihypertensive [18], anticancer [19-22], inhibition of blood platelet aggregation [23], antidepressant and anxiolytic [24,25], antioxidant [26] and antifungal [27]. Thus, the extensive biological activities of pyridine and pyridazine make them important in the design of drug-like molecules. Encouraged by the afore-mentioned findings and in a continuation of an ongoing program aimed at finding new structural leads with potential potent antibacterial and antifungal agents [28,29], this study describes the synthesis of a new class of 2-amino-5-arylazo-6-aryl substituted nicotinic acid, pyridazinone, and pyrido[2,3-*d*]pyrimidine derivatives.

## Results and discussion

### Synthetic chemistry

**Scheme 1:**
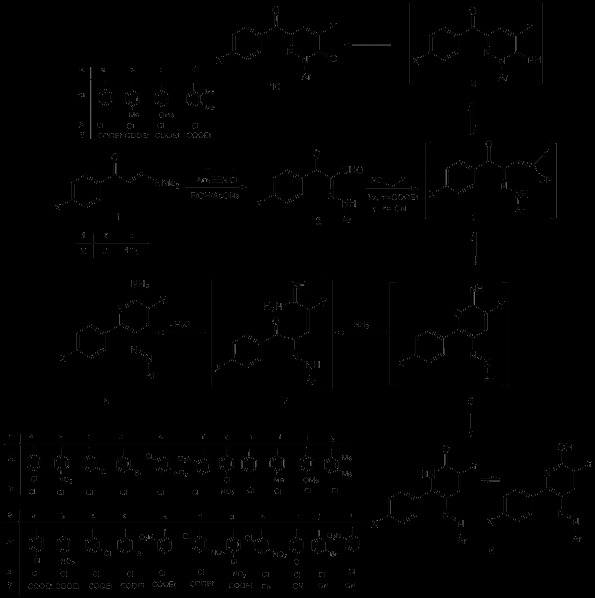
Synthesis of 2-amino-5-arylazonicotinic acid 8 and pyridazinone derivatives 10.

**Figure 1:**
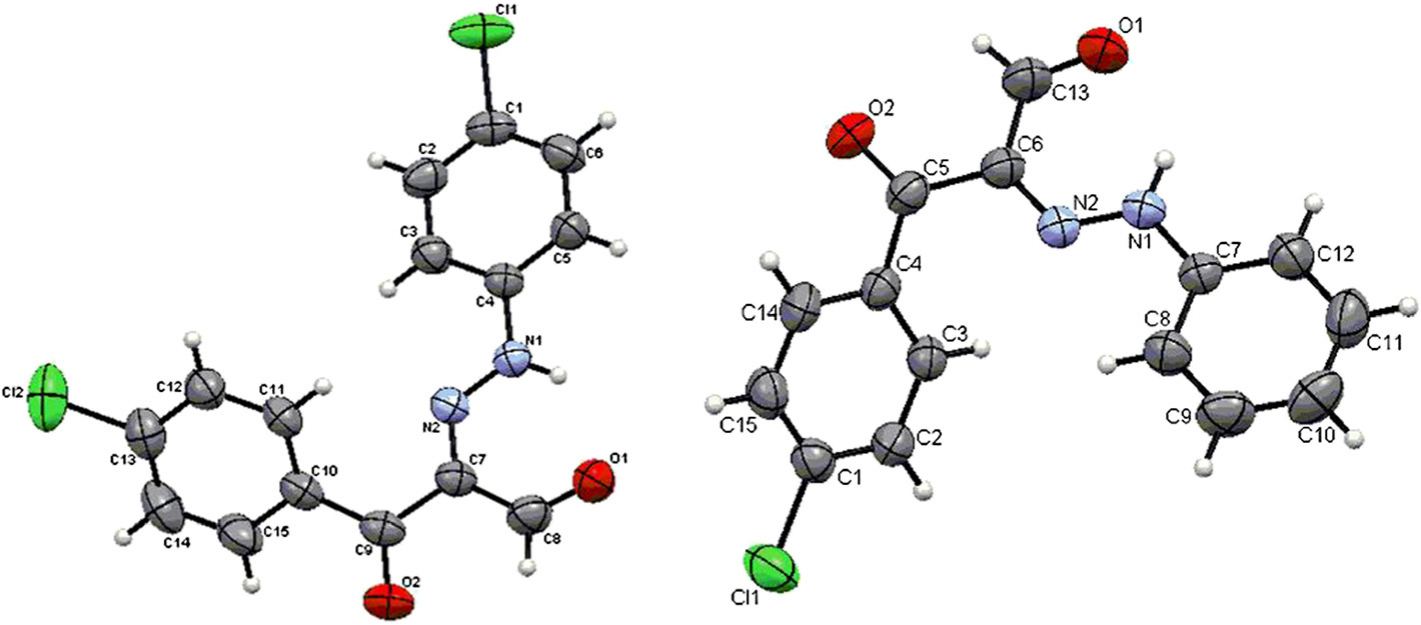
ORTEP plot of the X-ray crystallographic data determined for 2a and 2h.

**Figure 2:**
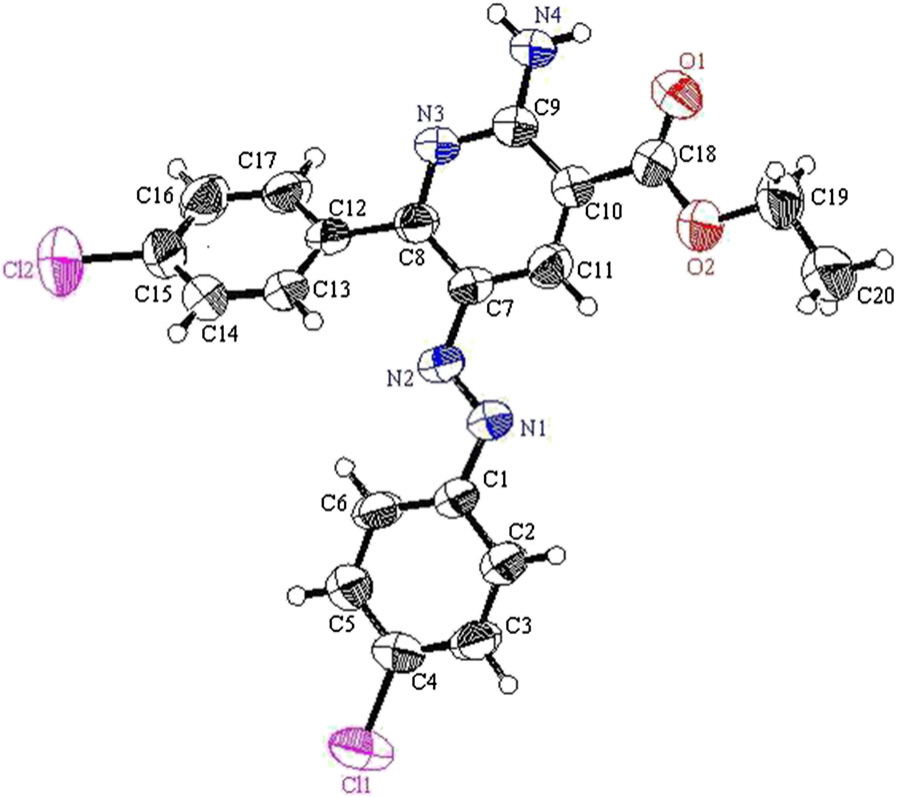
ORTEP plot of the X-ray crystallographic data determined for 8a.

**Figure 3:**
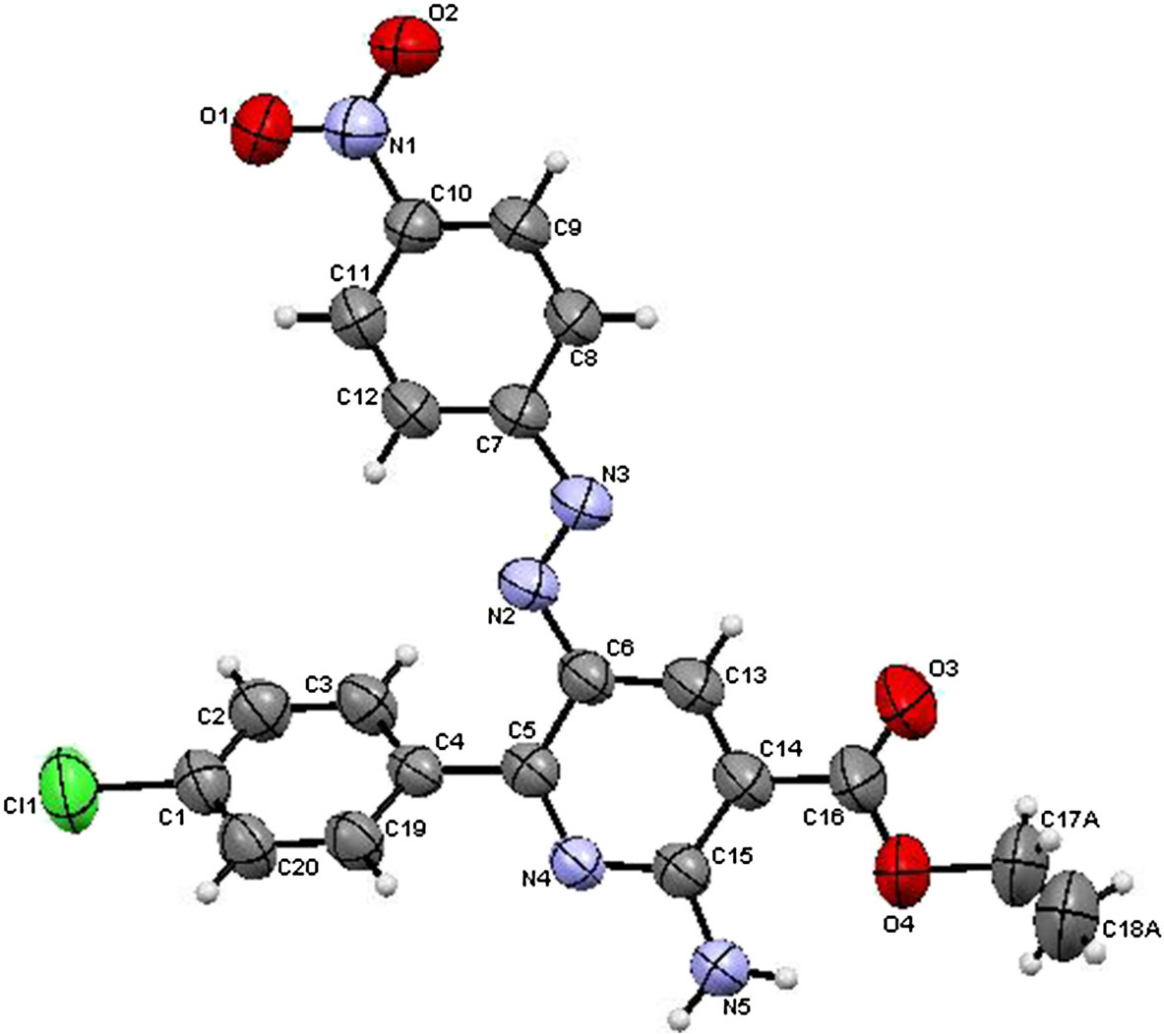
ORTEP plot of the X-ray crystallographic data determined for 8b.

**Figure 4:**
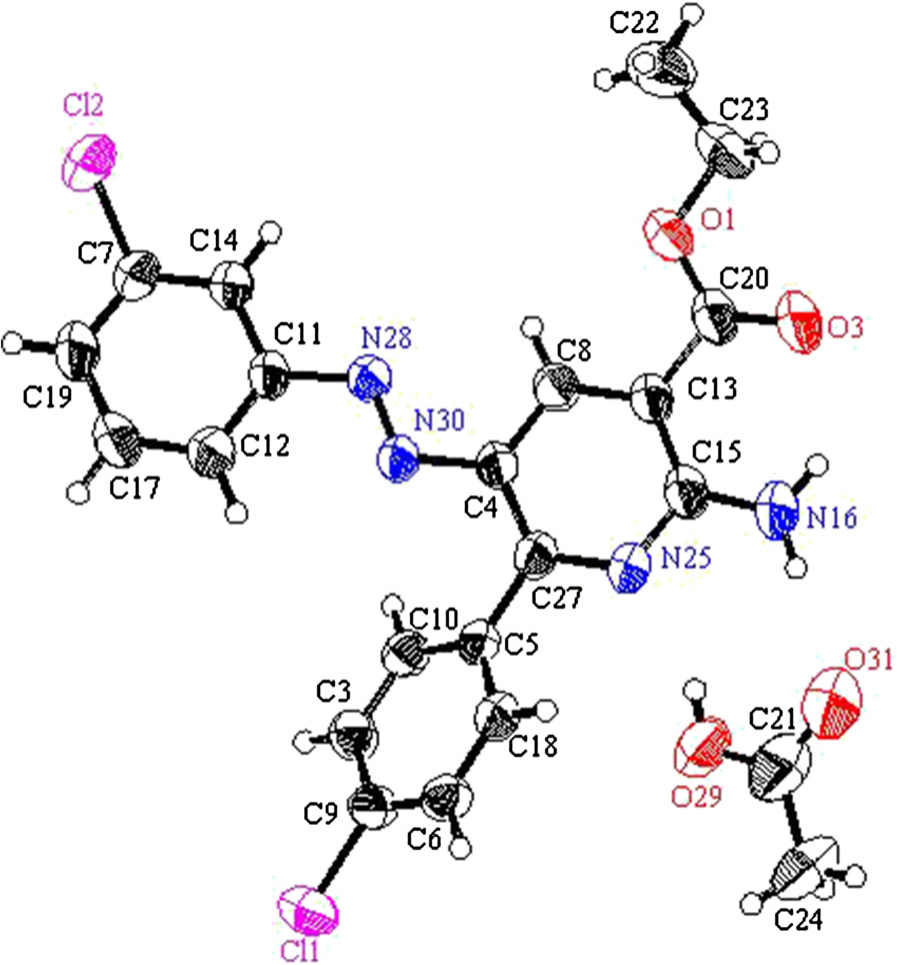
ORTEP plot of the X-ray crystallographic data determined for 8c.

**Figure 5:**
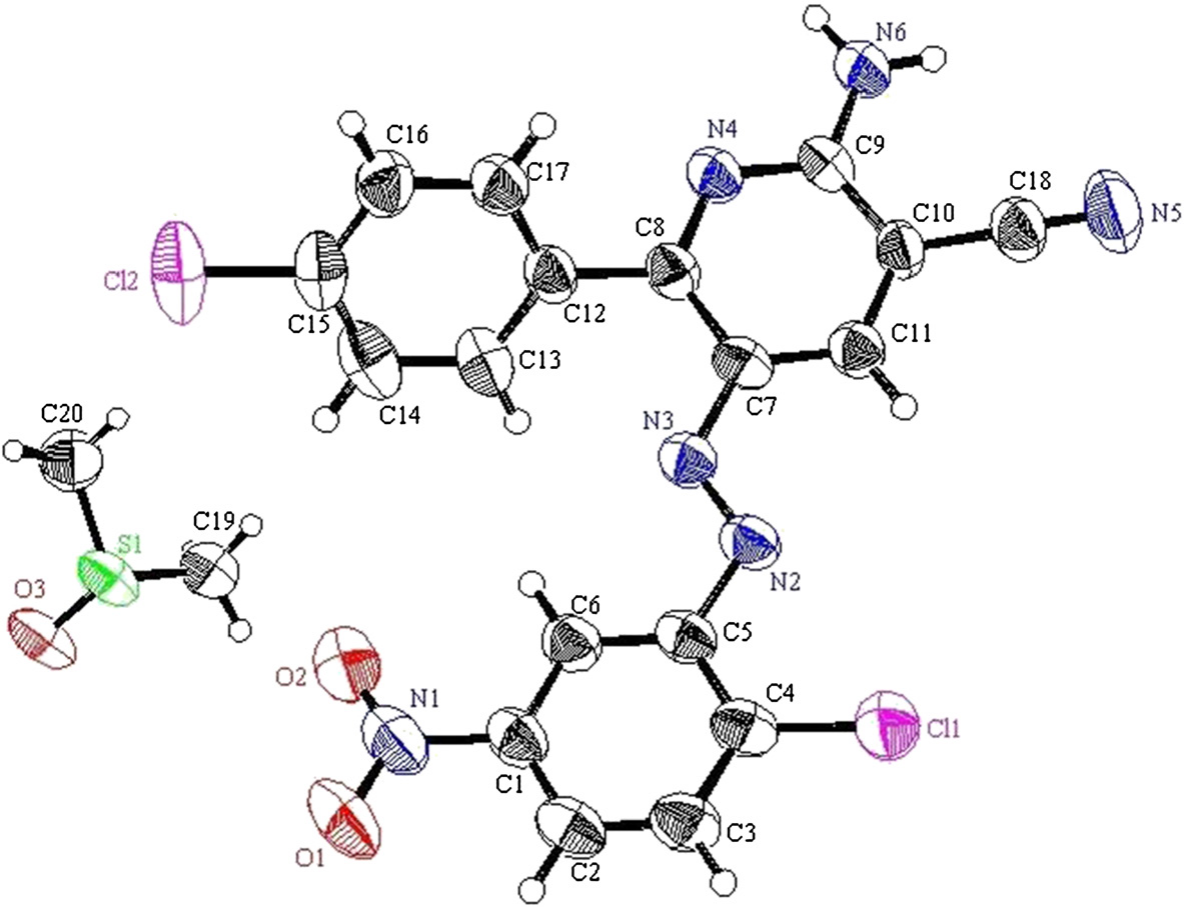
ORTEP plot of the X-ray crystallographic data determined for 8h.

**Figure 6:**
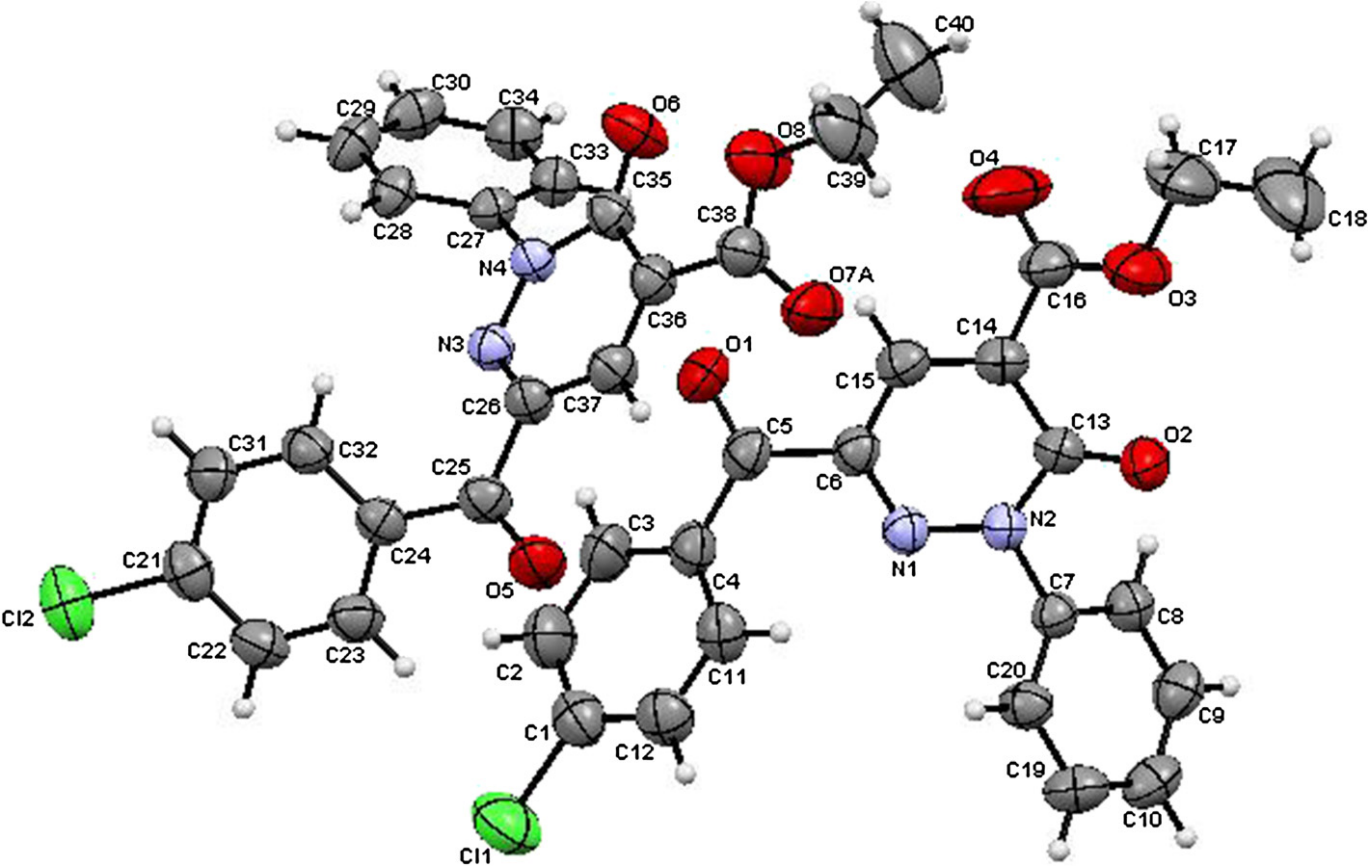
ORTEP plot of the X-ray crystallographic data determined for 10a.

The reaction of the 3-oxo-2-arylhydrazonopropanals **2** with the active methylene reagents has been investigated in the past [30]. Recently, it was shown that this reaction affords either arylazo-2-oxonicotinates **6** or pyridazinones **10**[31]. However, the factors that control the nature of the end product could not be defined. In the present article, we report the synthesis of several derivatives of **2** with electron-donating and -withdrawing substituents on the arylazo moiety and identified the exact structure of the products of their reaction with the active methylene reagents **3a,b**. It could be concluded that the reaction of **3** with **2** having an electron-donating substituent on the arylazo moiety afforded only the pyridazinones **10** while reacting **3** with **2** having an electron-withdrawing substituent on the arylazo moiety either in the *p*, *m,* or *o* position or a mix of them affords only the 2-amino-5-arylazo-6-aryl substituted nicotinic acid derivatives **8**. Thus compounds **2a-k** were prepared *via* coupling of **1** with aromatic diazonium salts [30] (cf. Scheme 1 and Figure 1). Reacting **2a-g** with ethyl cyanoacetate **3a** or with malononitrile **3b** affords the 2-amino-5-arylazo-6-aryl substituted nicotinates **8a-k** as confirmed from accurate mass determination and elemental analyses. Moreover, the structures were also confirmed from the X-ray single crystal structure determination for **8a**, **8b**, **8c**, and **8h** (cf. Figures 2, 3, 4, and 5, Tables 1, 2, and Scheme 1). It is believed that initially the acyclic condensation products **4** were formed and then these cyclize to the pyranimine **5** that reacts with ammonia from the reaction medium to yield the acyclic intermediate **7** that further cyclizes into the final isolable 2-aminonicotinic acid derivatives **8**. Under these conditions, no traces of the arylazo-2-oxonicotinates **6** or 2-hydroxy-5-arylazonicotinates were isolated as reported by Al-Mousawi *et al.*[31,32]. On the other hand, the reaction of **2h-k** having a phenyl or a phenyl substituted with an electron-donating group on the arylhydrazone moiety with **3a** afforded the pyridazinones **10a-d**. It is believed that also in this case, the acyclic intermediate **4** was formed and then cyclized *via* attack of the arylhydrazone moiety at CN to afford the pyridazine imine intermediate **9** that was hydrolyzed under the reaction conditions to yield the final isolable pyridazinone **10**. The structure of **10** was also supported by both the classical analytical analyses and through the X-ray crystal structure determination for **10a** (cf. Figure 6, Table 3, and Scheme 1). It is believed that the basicity of the hydrazone moiety of **2** controls the nature of the final product as it facilitates the reversible cyclization of the intermediate **4** and at the same time helps to stabilize the cyclized **9,** thus allowing the hydrolysis step to proceed to form the pyridazinone **10**. In contrast, cyclization of **4** is highly reversible and a competing cyclization reaction takes place resulting in formation of the pyranimine **5**, which in the presence of ammonium ion led to the formation of the stable aromatic 2-aminonicotinic acid derivatives **8**.

**Table 1:** Selected bond lengths and bond angles for 8a

**Bond**	**Bond length(Å)**	**Bond**	**Bond angle(o)**
N3-C8	1.332	C8-N3-C9	119.4
N3-C9	1.363	N3-C8-C7	122.5
N4-C9	1.327	N3-C8-C12	114.5
C9-C10	1.421	N3-C9-N4	116.3
N1-C1	1.427	N4-C9-C10	122.5
N1-N2	1.259	N1-N2-C7	116.0
N2-C7	1.406	N2-C7-C8	116.1

**Table 2:** Selected bond lengths and bond angles for 8h

**Bond**	**Bond length(Å)**	**Bond**	**Bond angle(o)**
N4-C8	1.344	C8-N4-C9	119.6
N4-C9	1.352	N4-C9-C10	120.8
N6-C9	1.342	N4-C8-C7	121.3
C9-C10	1.422	N4-C9-N6	116.9
C10-C18	1.433	N6-C9-C10	122.3
N5-C18	1.148	C9-C10-C18	119.6
N2-N3	1.254	N5-C18-C10	179.2
N3-C7	1.412	N2-N3-C7	115.2

**Table 3:** Selected bond lengths and bond angles for 10a

**Bond**	**Bond length(Å)**	**Bond**	**Bond angle(o)**
N1-N2	1.346	N1-N2-C13	126.04
N1-C6	1.312	N1-N2-C7	114.44
N2-C13	1.413	C13-N2-C7	119.32
O2-C13	1.217	N2-C13-C14	112.98
O1-C5	1.220	C6-N1-N2	117.37
N2-C7	1.442	O3-C16-C14	116.3

**Scheme 2:**
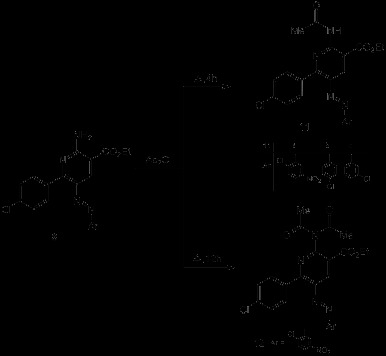
Synthesis of acylated azonicotinate derivatives 11 and 12.

**Figure 7:**
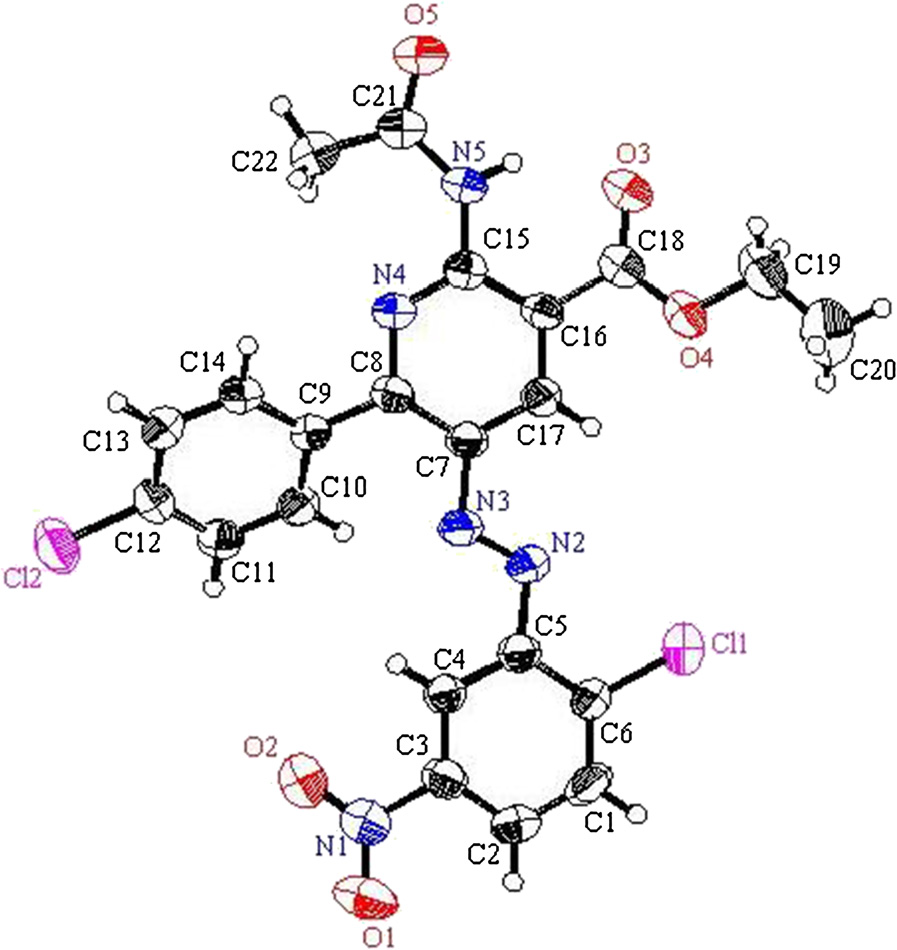
ORTEP plot of the X-ray crystallographic data determined for 11a.

**Figure 8:**
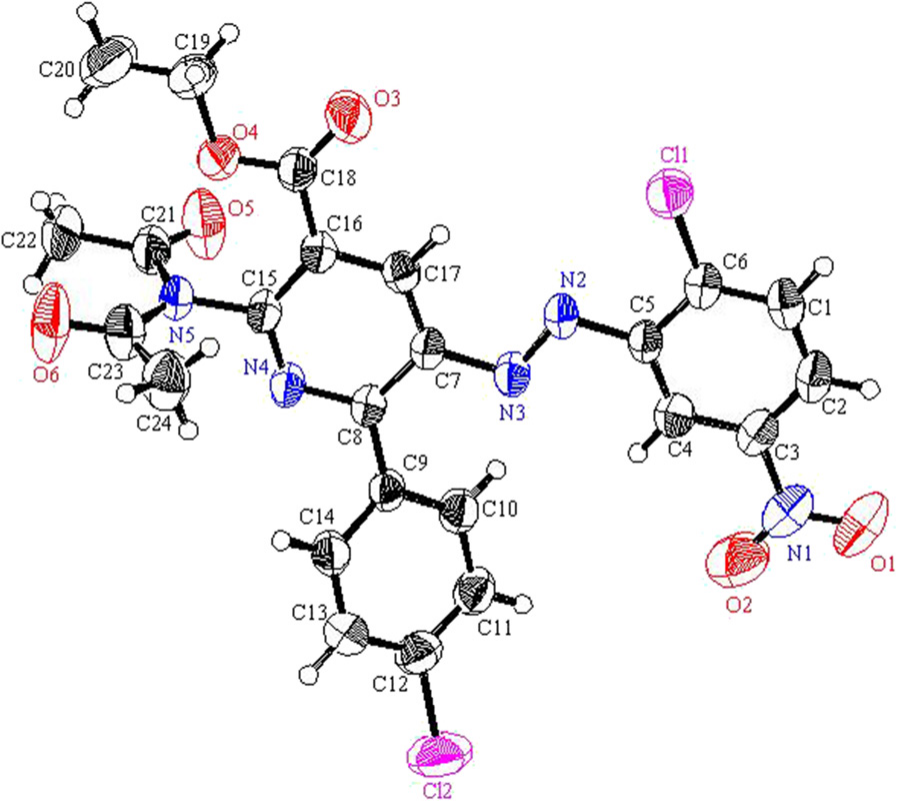
ORTEP plot of the X-ray crystallographic data determined for 12.

The obtained arylazoaminonicotinates are interesting precursors for the synthesis of a variety of a novel arylazoheterocycles that may possess interesting biological activities. Reaction of the 2-amino-5-arylazonicotinates **8** with acetic anhydride afforded the mono- and the di-acetylated products **11** and **12,** respectively, depending upon the reaction time. The structures of the products **11a** and **12** were confirmed by X-ray single crystal determination (cf. Scheme 2, Figures 7, 8).

**Scheme 3:**
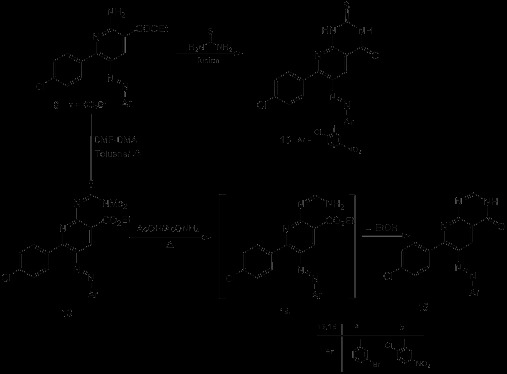
**Synthesis of pyrido[2,3-*d*]pyrimidine derivatives 15 and 16.**

Moreover, the 2-amino-5-arylazonicotinates **8** reacted with dimethylformamide dimethylacetal (DMF-DMA) to yield the corresponding amidines **13**. The amidines **13a,b** reacted with ammonia in refluxing acetic acid to yield the corresponding pyrido[2,3-*d*]pyrimidine derivatives **15a,b**. The structures of these products were also confirmed by different spectroscopic analyses as illustrated in the experimental section. Furthermore, fusion of the azonicotinates **8f** with thiourea afforded the corresponding pyrido[2,3-*d*]pyrimidine derivatives **16** (cf. Scheme 3).

## Antimicrobial activity

The novel chemical compounds synthesized in this study showed promising antimicrobial activities. In general, most of the tested compounds revealed better activity against Gram-positive rather than the Gram-negative bacteria and yeast. The results as depicted in Table 4 show strong activities against Gram-positive bacteria because all of the tested chemicals showed highly positive antimicrobial activities against *B. subtilis* with inhibition zones >10 mm. Only the tested chemical **1a** displayed strong inhibitory effects on the growth of *Escherichia coli* (Gram-negative bacteria), *Bacillus subtilis*, and *Staphylococcus aureus* (Gram-positive bacteria), which showed inhibition zones exceeding 10 mm. It also strongly inhibited the growth of *Candida albicans* (yeast) while the cycloheximide did not inhibit growth of this yeast. None of the chemicals except **1a** inhibited the growth of Gram-negative bacteria or yeast. Moreover compounds **2a**, **2c**, **2d**, and **2g** had high inhibitory activities against the Gram-positive bacteria *S. aureus.* The tested chemicals **8a-h** and **10a-c** displayed very strong inhibitory effects toward the growth of the Gram-positive bacteria *B. subtilis* with inhibition zones exceeding the reference chemotherapeutic ampicillin (cf. Table 4). Compounds **8a** and **10c** were also nearly as active as ampicillin against *B. subtilis* (MIC = 12.5 μg/mL). It was found that transformation of the enaminones **1** into the corresponding arylhydrazonals **2** generally decreased the inhibitory effects while transformation of the latter into the corresponding 2-amino-5-arylazo-6-aryl substituted nicotinates **8** or the pyridazinone **10** resulted in inhibition of the growth of only *B. subtilis* (Gram-positive bacteria) as revealed by the diameters of their inhibition zones. Conversely, conversion of the 2-aminoazonicotinates derivatives into the corresponding acetyl, diacetyl, or amidine derivatives exemplified by compounds **11**, **12**, and **13** unfortunately resulted in a decrease in the inhibitory effects but still had inhibition zones >10 mm. Fusing the pyridine ring into the bicyclic pyrido[2,3-*d*]pyrimidine derivatives **15a,b** and **16** enhanced the antimicrobial activity because the majority of these compounds were active against only the Gram-positive bacteria *B. subtilis* and *S. aureus*.

**Table 4:** Inhibition zone diameter (mm) of the tested chemicals that showed antimicrobial activities against the tested microorganisms

**Compound No.**	**Inhibition zone diameter in mm ± (standard deviation)**				
	***E. coli***	***P. aeruginosa***	***B. subtilis***	***S. aureus***	***C. albicans***
**1a**	20 (0.04)	0	23 (0.02)	12 (0.02)	34 (0.05)
**2a**	0	0	12 (0.04)	14 (0.10)	0
**2c**	0	0	12 (0.02)	10 (0.03)	0
**2d**	0	0	12 (0.03)	10 (0.07)	0
**2e**	0	0	11 (0.07)	8 (0.07)	0
**2g**	0	0	16 (0.04)	11 (0.05)	0
**8a**	0	0	30 (0.07)	0	0
**8b**	0	0	28 (0.10)	0	0
**8c**	0	0	23 (0.02)	0	0
**8d**	0	0	25 (0.09)	0	0
**8e**	0	0	29 (0.03)	0	0
**8f**	0	0	27 (0.10)	0	0
**8h**	0	0	26 (0.10)	0	0
**10a**	0	0	22 (0.20)	0	0
**10b**	0	0	25 (0.02)	0	0
**10c**	0	0	29 (0.20)	0	0
**11b**	0	0	13 (0.06)	6 (0.06)	
**12**	0	0	11(0.10)	4 (0.04)	
**13a**	0	0	16 (0.05)	0	0
**13b**	0	0	17 (0.10)	0	0
**15a**	0	0	19 (0.02)	12 (0.07)	0
**15b**	0	0	23 (0.03)	9 (0.2)	0
**16**	0	0	25 (0.09)	14 (0.02)	0
**DMSO (solvent)**	0	0	0	0	0
^a^**Ampicillin**	23 (0.14)	17 (0.07)	21 (0.05)	26 (0.07)	0
^b^**Cycloheximide**	-	-	-	-	0

DMSO = Dimethyl sulfoxide, ^a^ Ampicillin antibacterial drug, ^b^ cycloheximide antifungal drug, **–** not tested.

• Structure activity relationship

• By comparing the experimental biological activity of the compounds reported in this study with their structures, the following structural activity relationship assumptions are postulated.

➢ The pyridine or pyridazine moieties are necessary to observe the higher antibacterial activities towards the Gram-positive bacteria *B. subtilis*.

➢ It is interesting to point out that for the azonicotinates **8** having an electron-withdrawing group in the arylazo moiety in the *para-*, *meta*- and *ortho-* positions like compounds **8a-e** or having two electron-withdrawing groups in the arylazo moiety as in **8f** and **8h** results in higher antibacterial activity as evidenced by the inhibition zones that were similar (Table 4), and from the minimum inhibitory concentration (MIC) values presented in Table 5. This indicates that high antimicrobial activity may be correlated with the low electron density of the ring systems and the role of an electron-withdrawing group in increasing the antimicrobial potency is similar to the results of Sharma *et al.*[33].

**Table 5:** The MICs (μg/mL) of selected newly synthesized compounds against the tested microorganisms

**Compound No.**	**The minimum inhibitory concentration (MIC) in μg/mL**				
	***E. coli***	***P. aeruginosa***	***B. subtilis***	***S. aureus***	***C. albicans***
**1a**	50	-	50	-	12.5
**2a**	-	-	125	100	-
**2g**	-	-	100	-	-
**8a**	-	-	12.5	-	-
**8b**	-	-	25	-	-
**8c**	-	-	50	-	-
**8d**	-		25	-	
**8e**	-	-	25	-	-
**8g**	-	-	25	-	-
**10a**	-	-	50	-	-
**10b**	-	-	25	-	-
**10c**	-	-	12.5	-	-
**11b**	-	-	100	-	-
**13a**	-	-	100	-	-
**13b**	-	-	100	-	-
**15a**	-	-	100	-	-
**15b**	-	-	50	-	-
**16**	0	0	50	100	-
^a^**Ampicillin**	6.25	-	12.5	12.5	-
^b^**Cycloheximide**	-	-	-	-	-

^a^ Ampicillin antibacterial drug, ^b^ cycloheximide antifungal drug, – not measured.

➢ It is worth mentioning that changing the COOEt group to a CN group as in **8f** and **8h** has no significant effect on the biological activity.

➢ The presence of a Me or OMe (electron-donating group) in the aryl moiety in position 2 as in the pyridazine **10b,c** enhances the biological activity.

➢ Transformation of the azonicotinates **8** to the pyrido[2,3-*d*]pyrimidine derivatives **15a,b** and **16** does not significantly affect the biological activity against the Gram-positive bacteria *B. subtilis*.

## Experimental

### General

Melting points were recorded on a Griffin melting point apparatus and are reported uncorrected. IR spectra were recorded using KBr disks using a Perkin-Elmer System 2000 FT-IR spectrophotometer. ^1^H-NMR (400 MHz) or (600 MHz) and ^13^C-NMR (100 MHz) or (150 MHz) spectra were recorded at 25°C in CDCl_3_ or DMSO-*d*_*6*_ as solvent with TMS as internal standard on a Bruker DPX 400 or 600 super-conducting NMR spectrometer. Chemical shifts are reported in ppm. Mass spectra were measured using a high resolution GC-MS (DFS) thermo spectrometers with EI (70 EV). Microanalyses were performed on a LECO CHNS-932 Elemental Analyzer. Follow up of the reactions and checking homogeneity of the prepared compounds was made by thin layer chromatography (TLC). All single crystal data collections were made either on Rigaku R-AXIS RAPID diffractometer using Mo-Kα radiation (for samples **8a**, **8c**, **8h**, **11a** and **12**) or on Bruker X8 Prospector using Cu- Kα radiation (for compounds **2a**, **2h**, **8b**, and **10a**). The data were collected at room temperature. The structure was solved by direct methods and was expanded using Fourier techniques. The non-hydrogen atoms were refined anisotropically. In the case of compounds **8a**, **8c**, **8h**, **11a** and **12**, all calculations were performed using the Crystal Structure [34] crystallographic software package except for refinement, which was performed using SHELXL-97 [35]. In the case of **2a**, **2h**, **8b**, and **10a** the structure was solved and refined using the Bruker SHELXTL Software Package (Structure solution program- SHELXS-97 and Refinement program- SHELXL-97) [35] (cf. Additional files 1, 2, 3, 4, 5, 6, 7, 8, 9 and Table 6). Data were corrected for the absorption effects using the multi-scan method (SADABS). The enaminones **1a,b** and the arylhydrazonals **2a-k** were prepared according to the literature procedure [30,31].

**Table 6:** **The crystallographic data for the measured compounds**[36]

**Compound No.**	**Crystal Data**
**2a**	Clear light orange Block, C_15_H_10_Cl_2_N_2_O_2_, M = 321.16, triclinic, a = 6.3570(3) Å, b = 7.2377(3) Å, c = 16.7375(7) Å, V = 720.87(5) Å^3^, α = 80.359(3)°, β = 82.469(3)°, γ = 72.360(3)°, space group: P −1, Z = 2, D_calc_ = 1.480 g cm^−3^ , No. of reflection measured 2494, 2θ_max_ = 66.60°, R1 = 0.042.
**2h**	Clear light yellow Block, C_15_H_11_ClN_2_O_2_, M = 286.71, triclinic, a = 6.0147(2) Å, b = 7.3767(2) Å, c = 16.5968(4) Å, V = 672.60(3) Å^3^, α = 80.941(2)°, β = 85.3620(10)°, γ = 67.6940(10)°, space group: P −1, Z = 2, D_calc_ = 1.416 g cm^−3^ , No. of reflection measured 2324, 2θ_max_ = 66.74°, R1 = 0.036.
**8a**	Yellow platelet crystal, C_20_H_16_Cl_2_N_4_O_2_, M = 415.28, triclinic, a = 7.796(1) Å, b = 11.004(2) Å, c = 12.229(2) Å, V = 987.0(3) Å^3^, α = 70.789(8)°, β = 89.602(7)°, γ = 85.231(7)°, space group: P-1, Z = 2, D_calc_ = 1.397 g cm^−3^ , No. of reflection measured 3995, 2θ_max_ = 52.7°, R1 = 0.064.
**8b**	Clear light orange flake, C_20_H_16_ClN_5_O_4_, M = 425.83, monoclinic, a = 27.918(4) Å, b = 6.632(8) Å, c = 24.125(3) Å,V = 4082.0(9) Å^3^, α = γ = 90.00°, β = 113.965(9)°, space group: C 1 2/c 1, Z = 8, D_calc_ = 1.386 g cm^−3^ , No. of reflection measured 3434, 2θ_max_ = 66.59°, R1 = 0.082.
**8c**	Orange prism crystal, C_20_H_16_Cl_2_N_4_O_2_, M = 415.28, orthorhombic, a = 7.5481 (6) Å, b = 21.382(2) Å, c = 27.862(2) Å, V = 4496.8(6) Å^3^, α = β = γ = 90.0°, space group: Pbca, Z = 8, D_calc_ = 1.404 g cm^−3^ , No. of reflection measured 3923, 2θ_max_ = 50.0°, R1 = 0.067.
**8h**	Yellow block crystal, C_18_H_10_Cl_2_N_6_O_2_, M = 413.23, triclinic, a = 8.918(1) Å, b = 10.696(1) Å, c = 13.217(2) Å, V =1132.2(2) Å^3^, α = 73.044(6)°, β = 81.609(6)°, γ = 70.078(5)°, space group: P-1, Z = 2, D_calc_ = 1.441 g cm^−3^ , No. of reflection measured 4609, 2θ_max_ = 52.7°, R1 = 0.047.
**10a**	Clear light colorless block, C_20_H_15_ClN_2_O_4_, M = 382.81, monoclinic, a = 9.8702(7) Å, b = 18.7297(14) Å, c = 19.4912(15) Å, V = 3600.1(5) Å^3^, α = γ = 90°, β = 92.397(4)°, space group: P 1 21/c 1, Z = 8, D_calc_ = 1.412 g cm^−3^ , No. of reflection measured 6168, 2θ_max_ = 66.63°, R1 = 0.049.
**11**	Yellow needle crystal, C_22_H_17_Cl_2_N_5_O_5_, M = 502.32, orthorhombic, a = 26.252(2) Å, b = 7.3051(5) Å, c = 24.022(2) Å, V = 4606.8(6) Å^3^, α = β = γ = 90°, space group: Pbcn, Z = 8, D_calc_ = 1.448 g cm^−3^ , No. of reflection measured 4017, 2θ_max_ = 50.0°, R1 = 0.059.
**12**	Yellow needle crystal, C_24_H_19_Cl_2_N_5_O_6_, M = 544.35, monoclinic, a = 8.027(1) Å, b = 14.586(2) Å, c = 21.531(3) Å, V = 2499.1(5) Å^3^, α = γ = 90°, β = 97.548(7)°, space group: P21/c, Z = 4, D_calc_ = 1.447 g cm^−3^ , No. of reflection measured 5078, 2θ_max_ = 52.7°, R1 = 0.046.

#### General procedure for the preparation 2-amino-5-arylazo-6-aryl substituted nicotinates 8a-k

Independent mixtures of **2a-g** (10 mmol), active methylenenitrile derivatives **3a,b** (10 mmol), and ammonium acetate (2 g) in acetic acid (20 mL) were stirred at reflux for 1–2 h. (the progress of the reactions was monitored by using TLC using 1:1 ethyl acetate-petroleum ether as eluent). The mixtures were cooled and then poured into ice-water. The solids that so formed were collected by filtration and crystallized from the proper solvents to give **8a-k** as pure products.

#### 2-Amino-6-(4-chlorophenyl)-5-(4-chlorophenylazo) nicotinic acid ethyl ester (8a)

Recrystallized from an EtOH/dioxane (3:1) mixture as orange crystals, yield: (80%), m.p. 208–210°C; IR (KBr): *v* /cm^−1^ 3409, 3278 (NH_2_), 1699 (CO ester); ^1^H-NMR (DMSO-*d*_*6*_): δ = 1.35 (t, 3H, *J* = 7.2 Hz, *CH*_*3*_CH_2_), 4.37 (q, 2H, *J* = 7.2 Hz, CH_3_*CH*_*2*_), 7.57-7.63 (m, 4H, Ar-H), 7.73 (d, *J* = 8.4 Hz, 2H, Ar-H), 7.81 (d, *J* = 8.4 Hz, 2H, Ar-H), 7.88, 8.14 (two br, 2H, NH_2_, D_2_O exchangeable) and 8.58 ppm (s, 1H, pyridine H4); ^13^C-NMR (DMSO-*d*_*6*_): δ = 14.19 (CH_3_), 61.23 (CH_2_), 105.42 (pyridine C3), 124.12, 127.51, 127.71, 129.60, 132.75, 134.42, 135.02, 135.93, 136.38, 150.89, 159.79, 160.62 and 166.13 ppm (Ar-C and CO); MS (EI): m/z (%) 414 (M^+^, 100), 415 (M^+^+1, 70.85). Anal. calcd. for C_20_H_16_Cl_2_N_4_O_2_ (415.28): C, 57.85; H, 3.88; N, 13.49. Found: C, 57.93; H, 3.77; N, 13.57.

#### 2-Amino-6-(4-chlorophenyl)-5-(4-nitrophenylazo)nicotinic acid ethyl ester (8b)

Recrystallized from an EtOH/dioxane (2:1) mixture as deep orange crystals, yield: (85%), m.p. 230–231°C; IR (KBr): *v* /cm^−1^ 3402, 3297 (NH_2_), 1717 (CO ester); ^1^H-NMR (DMSO-*d*_*6*_): δ = 1.36 (t, 3H, *J* = 7.2 Hz, *CH*_*3*_CH_2_), 4.36 (q, 2H, *J* = 7.2 Hz, CH_3_*CH*_*2*_), 7.58 (d, *J* = 8.4 Hz, 2H, Ar-H), 7.80-7.86 (m, 4H, Ar-H), 7.98, 8.31 (two br, 2H, NH_2_, D_2_O exchangeable), 8.35 (d, *J* = 8.4 Hz, 2H, Ar-H) and 8.57 ppm (s, 1H, pyridine H4); ^13^C-NMR (DMSO-*d*_*6*_): δ = 14.11 (CH_3_), 61.25 (CH_2_), 105.60 (pyridine C3), 123.24, 125.03, 127.46, 127.70, 132.76, 134.58, 135.68, 136.65, 147.58, 155.58, 160.15, 161.64 and 165.94 ppm (Ar-C and CO); MS (EI): m/z (%) 425 (M^+^, 100), 426 (M^+^+1, 57.92). Anal. calcd. for C_20_H_16_ClN_5_O_4_ (425.83): C, 56.41; H, 3.79; N, 16.45. Found: C, 56.50; H, 3.72; N, 16.40.

#### 2-Amino-6-(4-chlorophenyl)-5-(3-chlorophenylazo)nicotinic acid ethyl ester (8c)

Recrystallized from acetic acid as orange crystals, yield: (76%), m.p. 188–190°C; IR (KBr): *v */cm^−1^ 3400, 3275 (NH_2_), 1688 (CO ester); ^1^H-NMR (DMSO-*d*_*6*_): δ = 1.35 (t, 3H, *J* = 7.2 Hz, *CH*_*3*_CH_2_), 4.35 (q, 2H, *J* = 7.2 Hz, CH_3_*CH*_*2*_), 7.52-7.57 (m, 4H, Ar-H), 7.61-7.66 (m, 2H, Ar-H), 7.78 (d, *J* = 8.4 Hz, 2H, Ar-H), 7.87, 8.15 (two br, 2H, NH_2_, D_2_O exchangeable) and 8.51 ppm (s, 1H, pyridine H4); ^13^C-NMR (DMSO-*d*_*6*_): δ = 14.60 (CH_3_), 61.65 (CH_2_), 105.85 (pyridine C3), 121.99, 122.14, 127.90, 128.03, 130.44, 131.57, 133.20, 134.48, 134.91, 136.31, 136.66, 153.70, 160.34, 161.26 and 166.51 ppm (Ar-C and CO); MS (EI): m/z (%) 414 (M^+^, 100), 415 (M^+^+1, 67.45). Anal. calcd. for C_20_H_16_Cl_2_N_4_O_2_ (415.28): C, 57.85; H, 3.88; N, 13.49. Found: C, 57.78; H, 3.94; N, 13.42.

#### 2-Amino-5-(3-bromophenylazo)-6-(4-chlorophenyl) nicotinic acid ethyl ester (8d)

Recrystallized from ethanol as deep yellow crystals, yield: (73%), m.p. 180–181°C; IR (KBr): *v* /cm^−1^ 3420, 3286 (NH_2_), 1697 (CO ester); ^1^H-NMR (DMSO-*d*_*6*_): δ = 1.35 (t, 3H, *J* = 7.2 Hz, *CH*_*3*_CH_2_), 4.35 (q, 2H, *J* = 7.2 Hz, CH_3_*CH*_*2*_), 7.48 (t, *J* = 8.0 Hz, 1H, Ar-H), 7.55 (d, *J* = 8.4 Hz, 2H, Ar-H), 7.64-7.70 (m, 2H, Ar-H), 7.75-7.80 (m, 3H, Ar-H), 7.88, 8.15 (two br, 2H, NH_2_, D_2_O exchangeable) and 8.51 ppm (s, 1H, pyridine H4); ^13^C-NMR (DMSO-*d*_*6*_): δ = 14.19 (CH_3_), 61.24 (CH_2_), 105.45 (pyridine C3), 122.19, 122.50, 124.44, 127.52, 127.63, 131.51, 132.80, 132.92, 134.49, 135.88, 136.24, 153.41, 159.93, 160.94 and 166.09 ppm (Ar-C and CO); MS (EI): m/z (%) 459 (M^+^, 100), 460 (M^+^+1, 69.55). Anal. calcd. for C_20_H_16_BrClN_4_O_2_ (459.73): C, 52.25; H, 3.51; N, 12.19. Found: C, 52.33; H, 3.45; N, 12.23.

#### 2-Amino-6-(4-chlorophenyl)-5-(2-nitrophenylazo)nicotinic acid ethyl ester (8e)

Recrystallized from an dioxane mixture as orange crystals, yield: (88%), m.p. 221–222°C; IR (KBr): *v*/cm^−1^ 3394, 3280 (NH_2_), 1704 (CO ester); ^1^H-NMR (DMSO-*d*_*6*_): δ = 1.33 (t, 3H, *J* = 7.2 Hz, *CH*_*3*_CH_2_), 4.35 (q, 2H, *J* = 7.2 Hz, CH_3_*CH*_*2*_), 7.50 (d, *J* = 8.0 Hz, 1H, Ar-H), 7.57 (d, *J* = 8.4 Hz, 2H, Ar-H), 7.67 (t, *J* = 8.0 Hz, 1H, Ar-H), 7.76-7.82 (m, 3H, Ar-H), 7.95, 8.31 (two br, 2H, NH_2_, D_2_O exchangeable), 8.05 (d, *J* = 8.0 Hz, 1H, Ar-H) and 8.47 ppm (s, 1H, pyridine H4); ^13^C-NMR (DMSO-*d*_*6*_): δ = 14.07 (CH_3_), 61.26 (CH_2_), 105.69 (pyridine C3), 118.66, 124.14, 127.78, 128.04, 130.73, 132.66, 133.57, 134.58, 135.68, 136.81, 144.31, 147.05, 160.12, 161.46 and 165.90 ppm (Ar-C and CO); MS (EI): m/z (%) 425 (M^+^, 17.25), 426 (M^+^+1, 7.05). HRMS (EI): m/z calcd. for C_20_H_16_^35^ClN_5_O_4_ (M^+^) 425.0885, found 425.0881.

#### 2-Amino-5-(2-chloro-5-nitrophenylazo)-6-(4-chlorophenyl)nicotinic acid ethyl ester (8f)

Recryst- allized from DMF as deep orange crystals, yield: (89%), m.p. 266–268°C; IR (KBr): *v* /cm^−1^ 3378, 3281 (NH_2_), 1709 (CO ester); ^1^H-NMR (DMSO-*d*_*6*_): δ = 1.35 (t, 3H, *J* = 7.2 Hz, *CH*_*3*_CH_2_), 4.38 (q, 2H, *J* = 7.2 Hz, CH_3_*CH*_*2*_), 7.57 (d, *J* = 8.4 Hz, 2H, Ar-H), 7.85 (d, *J* = 8.4 Hz, 2H, Ar-H), 7.96-8.41 (m, 5H, 3Ar-H and NH_2_) and 8.63 ppm (s, 1H, pyridine H4); ^13^C-NMR (DMSO-*d*_*6*_, at 100°C): δ = 13.95 (*CH*_*3*_CH_2_), 61.24 (CH_3_*CH*_*2*_), 106.27 (pyridine C3), 112.67, 124.64, 127.54, 128.12, 131.94, 132.63, 134.75, 135.81, 137.06, 139.12, 147.22, 148.64, 160.30, 161.67 and 165.88 ppm (Ar-C and CO); MS (EI): m/z (%) 459 (M^+^, 100), 460 (M^+^+1, 71.22). Anal. calcd. for C_20_H_15_Cl_2_N_5_O_4_ (460.28): C, 52.19; H, 3.28; N, 15.22. Found: C, 52.23; H, 3.35; N, 15.19.

#### 2-Amino-5-(4-chlorophenylazo)-6-(4-nitrophenyl)nicotinic acid ethyl ester (8g)

Recrystallized from an EtOH/dioxane (2:1) mixture as orange crystals, yield: (74%), m.p. 205–206°C; IR (KBr): *v* /cm^−1^ 3410, 3311 (NH_2_), 1723 (CO ester); ^1^H-NMR (DMSO-*d*_*6*_): δ = 1.37 (t, 3H, *J* = 7.2 Hz, *CH*_*3*_CH_2_), 4.39 (q, 2H, *J* = 7.2 Hz, CH_3_*CH*_*2*_), 7.61(d, *J* = 8.4 Hz, 2H, Ar-H), 7.73(d, *J* = 8.4 Hz, 2H, Ar-H), 8.05 (d, *J* = 8.4 Hz, 2H, Ar-H), 7.92, 8.20 (two br, 2H, NH_2_, D_2_O exchangeable), 8.37 (d, *J* = 8.4 Hz, 2H, Ar-H) and 8.62 ppm (s, 1H, pyridine H4); ^13^C-NMR (DMSO-*d*_*6*_): δ = 14.62 (CH_3_), 61.78 (CH_2_), 106.68 (pyridine C3), 123.16, 124.65, 128.07, 130.04, 132.60, 135.64, 137.06, 143.94, 148.19, 151.27, 157.18, 160.23 and 166.44 ppm (Ar-C and CO); MS (EI): m/z (%) 425 (M^+^, 100), 426 (M^+^+1, 85.15). Anal. calcd. for C_20_H_16_ClN_5_O_4_ (425.83): C, 56.41; H, 3.79; N, 16.45. Found: C, 56.48; H, 3.85; N, 16.52.

#### 2-Amino-5-(2-chloro-5-nitrophenylazo)-6-(4-chlorophenyl)nicotinonitrile (8h)

Recrystallized from DMSO as reddish brown crystals, yield: (77%), m.p. above 300°C; IR (KBr): *v* /cm^−1^ 3489, 3379 (NH_2_), 2220 (CN), 1628(C=N); ^1^H-NMR (DMSO-*d*_*6*_): δ = 7.57 (d, *J* = 8.4 Hz, 2H, Ar-H), 7.81 (d, *J* = 8.4 Hz, 2H, Ar-H), 7.96 (d, *J* = 8.8 Hz, 1H, Ar-H), 8.10 (s, 1H, Ar-H), 8.17 (br, 2H, NH_2_, D_2_O exchangeable), 8.27 (d, *J* = 8.8 Hz, 1H, Ar-H) and 8.36 ppm (s, 1H, pyridine H4); ^13^C-NMR (DMSO-*d*_*6*_): δ = 90.85 (pyridine C3), 112.58, 115.83, 125.24, 127.69, 131.14, 132.13, 132.81, 134.92, 135.42, 136.15, 139.71, 146.97, 148.04, 160.51 and 161.67 ppm (CN and Ar-C); MS (EI): m/z (%) 412 (M^+^, 100), 413 (M^+^+1, 61.45). Anal. calcd. for C_18_H_10_Cl_2_N_6_O_2_ (413.23): C, 52.32; H, 2.44; N, 20.34. Found: C, 52.25; H, 2.53; N, 20.40.

#### 2-Amino-6-(4-chlorophenyl)-5-(4-chlorophenylazo)nicotinonitrile (8i)

Recrystallized from dioxane as brown crystals, yield: (69%), m.p. 278–280°C; IR (KBr): *v* /cm^−1^ 3441, 3338 (NH_2_), 2213 (CN), 1656 (C=N); ^1^H-NMR (DMSO-*d*_*6*_): δ = 7.53-7.59 (m, 4H, Ar-H), 7.79 (d, *J* = 8.4 Hz, 2H, Ar-H), 7.84 (d, *J* = 8.4 Hz, 2H, Ar-H), 8.10 (br, 2H, NH_2_, D_2_O exchangeable) and 8.34 ppm (s, 1H, pyridine H4); MS (EI): m/z (%) 367 (M^+^, 74.75), 368 (M^+^+1, 29.50). Anal. calcd. for C_18_H_11_Cl_2_N_5_ (368.23): C, 58.71; H, 3.01; N, 19.02. Found: C, 58.62; H, 2.95; N, 19.11.

#### 2-Amino-5-(3-bromophenylazo)-6-(4-chlorophenyl)nicotinonitrile (8j)

Recrystallized from dioxane as brown crystals, yield: (72%), m.p. above 300°C; IR (KBr): *v* /cm^−1^ 3424, 3317 (NH_2_), 2208(CN), 1639(C=N); ^1^H-NMR (DMSO-*d*_*6*_): δ = 7.56-7.63 (m, 5H, Ar-H), 7.77-7.91 (m, 3H, 1 Ar-H and NH_2_), 7.97 (d, *J* = 8.4 Hz, 2H, Ar-H) and 8.22 ppm (s, 1H, pyridine H4); MS (EI): m/z (%) 412 (M^+^, 100), 413 (M^+^+1, 31.26). Anal. calcd. for C_18_H_11_BrClN_5_ (412.68): C, 52.39; H, 2.69; N, 16.97. Found: C, 52.47; H, 2.75; N, 17.05.

#### 2-Amino-6-(4-chlorophenyl)-5-(2-nitrophenylazo)nicotinonitrile (8k)

Recrystallized from a DMF/dioxane (1:1) mixture as yellowish brown crystals: (70%), m.p. 275–276°C; IR (KBr): *v* /cm^−1^ 3477, 3367 (NH_2_), 2222 (CN), 1635 (C=N); ^1^H-NMR (DMSO-*d*_*6*_): δ = 7.48 (d, *J* = 7.6 Hz, 1H, Ar-H), 7.57 (d, *J* = 8.0 Hz, 2H, Ar-H), 7.67 (t, *J* = 7.6 Hz, 1H, Ar-H), 7.77-7.79 (m, 3H, Ar-H), 8.04-8.10 (m, 3H, 1 Ar-H and NH_2_, D_2_O exchangeable), 8.16 ppm (s, 1H, pyridine H4); ^13^C-NMR (DMSO-*d*_*6*_): δ = 90.62 (pyridine C3), 115.89, 118.52, 124.17, 127.83, 131.02, 131.20, 132.65, 133.59, 134.79, 135.44, 136.42, 144.07, 147.18, 160.30 and 161.14 ppm (Ar-C and CN); MS (EI): m/z (%) 478 (M^+^, 100), 426 (M^+^+1, 46.55). HRMS (EI): m/z calcd. for C_18_H_11_^35^ClN_6_O_2_ (M^+^) 378.0626, found 378.0627.

#### General procedure for the preparation pyridazine derivatives 10a-d

Independent mixtures of **2h-k** (10 mmol), ethyl cyanoacetate **3a** (1.15 g, 10 mmol), and ammonium acetate (2 g) in acetic acid (20 mL) were stirred at reflux for 1–2 h. (the progress of the reactions was monitored by using TLC using 1:1 ethyl acetate-petroleum ether as eluent). The mixtures were cooled and then poured into iced water. The solids that so formed were collected by filtration and recrystallized from the proper solvents to give **10a-d** as pure products.

#### 6-(4-Chlorobenzoyl)-3-oxo-2-phenyl-2,3-dihydropyridazine-4-carboxylic acid ethyl ester (10a)

Recrystallized from an EtOH as yellow crystals, yield: (75%), m.p. 140–141°C; IR (KBr): *v* /cm^−1^ 1715, 1690 (CO and CO ester); ^1^H-NMR (DMSO-*d*_*6*_): δ = 1.32 (t, 3H, *J* = 7.2 Hz, *CH*_*3*_CH_2_), 4.35 (q, 2H, *J* = 7.2 Hz, CH_3_*CH*_*2*_), 7.46–7.55 (m, 3H, Ar-H), 7.60–7.62 (m, 4H, Ar-H), 8.04 (d, *J* = 8.4 Hz, 2H, Ar-H), and 8.33 ppm (s, 1H, pyridazine H5); ^13^C-NMR (DMSO-*d*_*6*_): δ = 13.97 (CH_3_), 61.86 (CH_2_), 125.94, 128.48, 128.87, 128.95, 131.06, 131.93, 132.41, 133.75, 138.38, 141.04, 141.27, 155.92, 162.60, and 187.71 ppm (Ar-C and CO); MS (EI): m/z (%) 382 (M^+^, 100), 383 (M^+^+1, 32.85). Anal. calcd. for C_20_H_15_ClN_2_O_4_ (382.81): C, 62.75; H, 3.95; N, 7.32. Found: C, 62.82; H, 3.92; N, 7.28.

#### 6-(4-Chlorobenzoyl)-3-oxo-2-*p*-tolyl-2,3-dihydropyridazine-4-carboxylic acid ethyl ester (10b)

Recrystallized from EtOH as yellow crystals, yield: (69%), m.p. 94–95°C; IR (KBr): *v*/cm^−1^ 1753, 1687 (CO and CO ester); ^1^H-NMR (DMSO-*d*_*6*_): δ = 1.31 (t, 3H, *J* = 7.2 Hz, *CH*_*3*_CH_2_), 2.37 (s, 3H, CH_3_), 4.35 (q, 2H, *J* = 7.2 Hz, CH_3_*CH*_*2*_), 7.33 (d, *J* = 8.4 Hz, 2H, Ar-H), 7.49 (d, *J* = 8.4 Hz, 2H, Ar-H), 7.62 (d, *J* = 8.4 Hz, 2H, Ar-H), 8.03 (d, *J* = 8.4 Hz, 2H, Ar-H) and 8.32 ppm (s, 1H, pyridazine H5); ^13^C-NMR (DMSO-*d*_*6*_): δ = 14.43 (CH_3_), 21.19 (CH_3_), 62.29 (CH_2_), 126.13, 128.91, 129.73, 131.41, 132.22, 132.85, 134.26, 138.75, 139.07, 139.10, 141.62, 156.38, 163.09 and 188.16 ppm (Ar-C and CO); MS (EI): m/z (%) 396 (M^+^, 100), 397 (M^+^+1, 34.52). Anal. calcd. for C_21_H_17_ClN_2_O_4_ (396.83): C, 63.56; H, 4.32; N, 7.06. Found: C, 63.63; H, 4.26; N, 7.15.

#### 6-(4-Chlorobenzoyl)-2-(4-methoxyphenyl)-3-oxo-2,3-dihydropyridazine-4-carboxylic acid ethyl ester (10c)

Recrystallized from EtOH as yellow crystals, yield: (72%), m.p. 132–133°C; IR (KBr): *v* /cm^−1^ 1757, 1679 (CO and CO ester); ^1^H-NMR (DMSO-*d*_*6*_): δ = 1.32 (t, 3H, *J* = 7.2 Hz, *CH*_*3*_CH_2_), 3.81 (s, 3H, OCH_3_), 4.35 (q, 2H, *J* = 7.2 Hz, CH_3_*CH*_*2*_), 7.06 (d, *J* = 8.8 Hz, 2H, Ar-H), 7.53 (d, *J* = 8.8 Hz, 2H, Ar-H), 7.62 (d, *J* = 8.4 Hz, 2H, Ar-H), 8.03 (d, *J* = 8.4 Hz, 2H, Ar-H) and 8.31 ppm (s, 1H, pyridazine H5); ^13^C-NMR (DMSO-*d*_*6*_): δ = 14.43 (CH_3_), 55.94 (CH_3_), 62.28 (CH_2_), 114.37, 127.63, 128.92, 131.28, 132.15, 132.86, 134.29, 134.40, 138.74, 141.51, 156.46, 159.75, 163.14 and 188.22 ppm (Ar-C and CO); MS (EI): m/z (%) 412 (M^+^, 100), 413 (M^+^+1, 28.95). Anal. calcd. for C_21_H_17_ClN_2_O_5_ (412.83): C, 61.10; H, 4.15; N, 6.79. Found: C, 61.17; H, 4.21; N, 6.75.

#### 6-(4-Chlorobenzoyl)-2-(2,3-dimethylphenyl)-3-oxo-2,3-dihydropyridazine-4-carboxylic acid ethyl ester (10d)

Recrystallized from EtOH as pale yellow crystals, yield: (70%), m.p. 96–97°C; IR (KBr): *v* /cm^−1^ 1746, 1692 (CO and CO ester); ^1^H-NMR (DMSO-*d*_*6*_): δ = 1.33 (t, 3H, *J* = 7.2 Hz, *CH*_*3*_CH_2_), 2.00 (s, 3H, CH_3_), 2.31 (s, 3H, CH_3_), 4.35 (q, 2H, *J* = 7.2 Hz, CH_3_*CH*_*2*_), 7.24-7.31 (m, 3H, Ar-H), 7.58 (d, *J* = 8.4 Hz, 2H, Ar-H), 7.95 (d, *J* = 8.4 Hz, 2H, Ar-H) and 8.38 ppm (s, 1H, pyridazine H5); MS (EI): m/z (%) 410 (M^+^, 100), 411 (M^+^+1, 35.14). Anal. calcd. for C_22_H_19_ClN_2_O_4_ (410.86): C, 64.32; H, 4.66; N, 6.82. Found: C, 64.36; H, 4.73; N, 6.89.

#### General procedure for the preparation compounds 11a-c and 12

Independent solutions of the azonicotinates **8a**,**c**,**f** (10 mmol) in acetic anhydride (10 mL) were stirred at reflux for 4 h. in case of compounds **11a-c** and for 12 h. in case of compound **12**. Then, the reaction mixture was allowed to cool to room temperature, the formed crude product was collected by filtration washed with ethanol and recrystallized from the proper solvent.

#### 2-Acetylamino-5-(2-chloro-5-nitrophenylazo)-6-(4-chlorophenyl)nicotinic acid ethyl ester (11a)

Recrystallized from EtOH/dioxane (2:1) mixture as reddish orange crystals, yield: (83%), m.p. 257–258°C; IR (KBr): *v* /cm^−1^ 3231 (NH), 1719, 1673 (2 CO); ^1^H-NMR (DMSO-*d*_*6*_): δ = 1.30 (t, 3H, *J* = 7.2 Hz, *CH*_*3*_CH_2_), 2.23 (s, 3H, CO*CH*_*3*_), 4.29 (q, 2H, *J* = 7.2 Hz, CH_3_*CH*_*2*_), 7.64 (d, *J* = 8.4 Hz, 2H, Ar-H), 7.91 (d, *J* = 8.4 Hz, 2H, Ar-H), 8.06 (d, *J* = 8.4 Hz, 1H, Ar-H), 8.20 (s, 1H, Ar-H), 8.39 (d, *J* = 8.4 Hz, 1H, Ar-H), 8.43 (s, 1H, pyridine H4) and 11.22 ppm (s, 1H, NH); ^13^C-NMR (DMSO-*d*_*6*_): δ = 14.49 (CH_3_), 24.38 (CH_3_), 61.78 (CH_2_), 113.36, 119.16, 126.98, 128.44, 129.16, 131.98, 132.11, 132.78, 133.57, 135.32, 135.57, 141.21, 147.48, 148.45, 158.16, 165.63 and 170.34 ppm (Ar-C and CO); MS (EI): m/z (%) 501 (M^+^, 100), 502 (M^+^+1, 72.45). Anal. calcd. for C_22_H_17_Cl_2_N_5_O_5_ (502.32): C, 52.61; H, 3.41; N, 13.94. Found: C, 52.64; H, 3.37; N, 13.88.

#### 2-Acetylamino-6-(4-chlorophenyl)-5-(4-chlorophenylazo)nicotinic acid ethyl ester (11b)

Recrys- tallized from EtOH/dioxane (3:1) mixture as orange crystals, yield: (77%), m.p. 103–104°C; IR (KBr): *v* /cm^−1^ 3278 (NH), 1721, 1685 (CO and CO ester); ^1^H-NMR (DMSO-*d*_*6*_): δ = 1.30 (t, 3H, *J* = 7.2 Hz, *CH*_*3*_CH_2_), 2.21 (s, 3H, CO*CH*_*3*_), 4.28 (q, 2H, *J* = 7.2 Hz, CH_3_*CH*_*2*_), 7.63 (d, *J* = 8.4 Hz, 2H, Ar-H), 7.69 (d, *J* = 8.4 Hz, 2H, Ar-H), 7.85-7.88 (m, 4H, Ar-H), 8.40 (s, 1H, pyridine H4) and 11.10 ppm (s, 1H, NH); MS (EI): m/z (%) 456 (M^+^, 76.92), 457 (M^+^+1, 67.22). Anal. calcd. for C_22_H_18_Cl_2_N_4_O_3_ (457.32): C, 57.78; H, 3.97; N, 12.25. Found: C, 57.86; H, 3.88; N, 12.27.

#### 2-Acetylamino-6-(4-chlorophenyl)-5-(3-chlorophenylazo)nicotinic acid ethyl ester (11c)

Recrystallized from EtOH/dioxane (3:1) mixture as orange crystals, yield: (79%), m.p. 210–211°C; IR (KBr): *v* /cm^−1^ 3245 (NH), 1709, 1680(2 CO); ^1^H-NMR (DMSO-*d*_*6*_): δ = 1.30 (t, 3H, *J* = 7.2 Hz, *CH*_*3*_CH_2_), 2.21 (s, 3H, CO*CH*_*3*_), 4.28 (q, 2H, *J* = 7.2 Hz, CH_3_*CH*_*2*_), 7.62-7.66 (m, 4H, Ar-H), 7.81-7.87 (m, 4H, Ar-H), 8.39 (s, 1H, pyridine H4) and 11.11 ppm (s, 1H, NH); ^13^C-NMR (DMSO-*d*_*6*_): δ = 14.04 (CH_3_), 23.91 (CH_3_), 61.27 (CH_2_), 122.17, 124.06, 126.98, 127.98, 131.40, 132.93, 134.21, 134.86, 135.02, 136.95, 139.80, 140.70, 142.90, 150.33, 156.69, 165.26 and 169.75 ppm (Ar-C and CO); MS (EI): m/z (%) 456 (M^+^, 91.14), 457 (M^+^+1, 85.08). Anal. calcd. for C_22_H_18_Cl_2_N_4_O_3_ (457.32): C, 57.78; H, 3.97; N, 12.25. Found: C, 57.69; H, 3.93; N, 12.31.

#### 5-(2-Chloro-5-nitrophenylazo)-6-(4-chlorophenyl)-2-diacetylaminonicotinic acid ethyl ester (12)

Recrystallized from EtOH as red crystals, yield: (89%), m.p. 229–230°C; IR (KBr): *v*/cm^−1^ 3231 (NH), 1720, 1705, 1681 (3 CO); ^1^H-NMR (DMSO-*d*_*6*_): δ = 1.31 (t, 3H, *J* = 7.2 Hz, *CH*_*3*_CH_2_), 2.31 (s, 6H, 2CO*CH*_*3*_), 4.36 (q, 2H, *J* = 7.2 Hz, CH_3_*CH*_*2*_), 7.64 (d, *J* = 8.4 Hz, 2H, Ar-H), 7.91 (d, *J* = 8.4 Hz, 2H, Ar-H), 8.10 (d, *J* = 8.4 Hz, 1H, Ar-H), 8.23 (s, 1H, Ar-H), 8.45 (d, *J* = 8.4 Hz, 1H, Ar-H) and 8.64 ppm (s, 1H, pyridine H4); ^13^C-NMR (DMSO-*d*_*6*_): δ = 14.34 (CH_3_), 26.75 (CH_3_), 62.67 (CH_2_), 113.56, 124.98, 127.68, 128.44, 128.73, 129.79, 132.93, 133.57, 133.76, 134.51, 136.14, 141.32, 144.73, 147.51, 148.43, 152.96, 158.34, 163.37 and 172.37 ppm (Ar-C and CO); MS (EI): m/z (%) 543 (M^+^, 8.55), 544 (M^+^+1, 2.95). Anal. calcd. for C_24_H_19_Cl_2_N_5_O_6_ (544.35): C, 52.96; H, 3.52; N, 12.87. Found: C, 53.02; H, 3.45; N, 12.94.

#### General procedure for the preparation of amidines 13a-b

Independent mixtures of the 2-amino-5-arylazonicotinates **8d,f** (5 mmol), N,N-dimethylformamide dimethylacetal (DMF-DMA) (0.6 mL, 5 mmol) in dry toluene (20 mL) were stirred at reflux for 4 h. The separated solid product obtained on standing at room temperature was collected by filtration, washed by EtOH and recrystallized from dioxane to afford the corresponding amidines **13a,b** as pure products.

#### 5-(3-Bromophenylazo)-6-(4-chlorophenyl)-2-(dimethyl-aminomethyleneamino)nicotinic acid ethyl ester (13a)

reddish brown crystals, yield: (72%), m.p. 226.-227°C; IR (KBr): *v* /cm^−1^ 1727 (CO ester); ^1^H-NMR (DMSO-*d*_*6*_): δ = 1.33 (t, 3H, *J* = 7.2 Hz, *CH*_*3*_CH_2_), 3.10 (s, 3H, CH_3_), 3.21 (s, 3H, CH_3_), 4.34 (q, 2H, *J* = 7.2 Hz, CH_3_*CH*_*2*_), 7.56-770 (m, 4H, Ar-H), 7.77-7.88 (m, 4H, Ar-H), 8.21 (s, 1H, amidine H) and 8.75 ppm (s, 1H, pyridine H4); MS (EI): m/z (%) 514 (M^+^, 100), 515 (M^+^+1, 42.57). Anal. calcd. for C_23_H_21_BrClN_5_O_2_ (514.81): C, 53.66; H, 4.11; N, 13.60. Found: C, 53.74; H, 4.15; N, 13.53.

#### 5-(2-Chloro-5-nitrophenylazo)-6-(4-chlorophenyl)-2-(dimethylaminomethyleneamino)nicotinic acid ethyl ester (13b)

red crystals, yield: (79%), m.p. 196–197°C; IR (KBr): *v* /cm^−1^ 1745 (CO ester); ^1^H-NMR (DMSO-*d*_*6*_): δ = 1.33 (t, 3H, *J* = 7.2 Hz, *CH*_*3*_CH_2_), 3.12 (s, 3H, CH_3_), 3.23 (s, 3H, CH_3_), 4.33 (q, 2H, *J* = 7.2 Hz, CH_3_*CH*_*2*_), 7.57 (d, *J* = 8.8 Hz, 2H, Ar-H), 7.91 (d, *J* = 8.8 Hz, 2H, Ar-H), 7.99 (d, *J* = 8.4 Hz, 1H, Ar-H), 8.16 (s, 1H, Ar-H), 8.23 (s, 1H, amidine H), 8.30 (d, *J* = 8.4 Hz, 1H, Ar-H) and 8.82 ppm (s, 1H, pyridine H4); ); ^13^C-NMR (DMSO-*d*_*6*_): δ = 14.52 (*CH*_*3*_CH_2_), 35.29 (CH_3_), 40.84 (CH_3_), 61.23 (CH_3_*CH*_*2*_), 113.22, 122.14, 125.54, 125.92, 128.08, 132.50, 133.30, 134.95, 136.80, 139.98, 140.08, 147.72, 149.14, 157.25, 158.55, 162.17 and 166.99 ppm (Ar-C and CO); MS (EI): m/z (%) 514 (M^+^, 100), 515 (M^+^+1, 68.14). Anal. calcd. for C_23_H_20_Cl_2_N_6_O_4_ (515.36): C, 53.60; H, 3.91; N, 16.31. Found: C, 53.57; H, 3.87; N, 16.24.

#### General Procedure for the Preparation of pyrido[2,3-*d*]pyrimidin-4-one 15a-b

Independent solutions of the amidines **13a,b** (5 mmol) in AcOH (20 mL) containing ammonium acetate (1.5 g) were stirred at reflux for 4 h. Then, the reaction mixtures were cooled to room temperature and poured onto ice cold water. The crude products were collected by filtration, washed with water and recrystallized from the appropriate solvent to afford the pyrido[2,3-*d*]pyrimidin derivatives **15a,b**.

#### 6-(3-Bromophenylazo)-7-(4-chlorophenyl)-3*H*-pyrido[2,3-*d*]pyrimidin-4-one (15a)

Reddish brown crystals, yield: (70%), m.p. 290–291°C; IR (KBr): *v* /cm^−1^ 3201 (NH), 1707 (CO); ^1^H-NMR (DMSO-*d*_*6*_): δ = 7.57-7.64 (m, 3H, Ar-H), 7.79-7.88 (m, 4H, Ar-H), 7.95 (s, 1H, Ar-H), 8.46 (s, 1H, pyrimidine H2), 8.63 (s, 1H, pyridine H5) and 12.78 ppm (s, 1H, NH) ; ^13^C-NMR (DMSO-*d*_*6*_): δ = 117.54, 122.58, 122.74, 123.05, 125.18, 127.98, 131.67, 133.01, 134.59, 134.99, 135.56, 143.13, 150.50, 153.10, 159.76, 160.48 and 161.66 ppm (Ar-C and CO); MS (EI): m/z (%) 440 (M^+^, 100), 441 (M^+^+1, 36.87). Anal. calcd. for C_19_H_11_BrClN_5_O (440.69): C, 51.79; H, 2.52; N, 15.89. Found: C, 51.87; H, 2.46; N, 15.92.

#### 6-(2-Chloro-5-nitrophenylazo)-7-(4-chlorophenyl)-3*H*-pyrido[2,3-*d*]pyrimidin-4-one (15b)

Reddish brown crystals, yield: (74%), m.p. above 300°C; IR (KBr): *v* /cm^−1^ 3212 (NH), 1702 ( CO); ^1^H-NMR (DMSO-*d*_*6*_): δ = 7.62 (d, *J* = 8.4 Hz, 2H, Ar-H), 7.87 (d, *J* = 8.4 Hz, 2H, Ar-H), 8.04 (d, *J* = 8.8 Hz, 1H, Ar-H), 8.16 (s, 1H, Ar-H), 8.37 (d, *J* = 8.8 Hz, 1H, Ar-H), 8.46 (s, 1H, pyrimidine H2), 8.66 (s, 1H, pyridine H5) and 12.74 ppm (s, 1H, NH); ^13^C-NMR (DMSO-*d*_*6*_): δ = 112.84, 117.23, 123.10, 126.31, 127.88, 132.31, 133.12, 134.84, 135.78, 135.93, 140.48, 142.44, 146.98, 148.06, 154.98, 158.82 and 160.94 ppm (Ar-C and CO); MS (EI): m/z (%) 440 (M^+^, 100), 441 (M^+^+1, 55.15). Anal. calcd. for C_19_H_10_Cl_2_N_6_O_3_ (441.24): C, 51.72; H, 2.28; N, 19.05. Found: C, 51.68; H, 2.34; N, 19.13.

#### 6-(2-Chloro-5-nitrophenylazo)-7-(4-chlorophenyl)2-thioxo-2,3-dihydro-1H-pyrido[2,3-d]pyrimidin-4-one (16)

A mixture of compound **8f** (1.15 g, 2.5 mmol), thiourea (0.2 g, 2.5 mmol), and a few drops from DMF was fused together in an oil bath under nitrogen gas at 250°C for 15 min, the fused mass was dissolved in DMF and poured onto ice cold water, the solid obtained was recrystallized from dioxane/DMF (2:1) to give compounds **16** as brown crystals, yield: (64%), m.p. above 300°C; IR (KBr): *v* /cm^−1^ 3326, 3264 (2NH), 1701 (CO); ^1^H-NMR (DMSO-*d*_*6*_): δ = 7.57 (d, *J* = 8.4 Hz, 2H, Ar-H), 7.85 (d, *J* = 8.4 Hz, 2H, Ar-H), 8.13 (d, *J* = 8.4 Hz, 1H, Ar-H), 8.24 (s, 1H, Ar-H), 8.41 (d, *J* = 8.4 Hz, 1H, Ar-H), 8.59 (s, 1H, pyridine H5), 9.17 (s, 1H, NH), and 12.45 ppm (s, 1H, NH); MS (EI): m/z (%) 472 (M^+^, 58.56), 473 (M^+^+1, 16.77). Anal. calcd. for C_19_H_10_Cl_2_N_6_O_3_S (473.30): C, 48.22; H, 2.13 N, 17.76; S, 6.77. Found: C, 48.10; H, 2.17; N, 17.65; S, 6.62.

## Antimicrobial evaluation

### Methodology

The antimicrobial activities of newly synthesized 22 different chemical compounds were tested using the Agar-well diffusion technique (Isaacson and Kirchbaum) [37] against five different microbial cultures. Pure cultures of *E. coli* (Culture *#*0680P, Microbiologics, St. Cloud, MN, USA) and *P. aeruginosa* (Culture #0416P, Microbiologics) (Gram-negative bacteria), *B. subtilis* (Culture #0269P, Microbiologics), and *S. aureus* (Culture #0831P, Microbiologics) (Gram-positive bacteria) and *C. albicans* (yeast) (Culture #155965, Carolina Biological Supply, Burlington, NC, USA) were involved in the test. Bacterial strain cultures were cultivated in Mueller–Hinton broth (Difco) for all the bacterial strains after 24 h of incubation at 37°C. The yeasts were propagated in Sabouraud dextrose broth (Difco) after incubation for 24 h at 25°C, [38,39] an aliquot of 0.1 ml of each bacterial strain was inoculated and spread on nutrient agar (NA), while 0.1 ml of the yeast was spread on potato dextrose agar (PDA). The inoculated plates were supplied with 100 μl of each of the tested chemicals with a total final concentration of 1 mg ml^-1^. The chemicals were included in 4-mm wells produced by a sterile cork borer. The NA plates were incubated at 37°C for 24 h while PDA plates were incubated at 25°C for 48 h. The zones of inhibition around the wells were determined and the average based on three replicas was recorded. Ampicillin and cycloheximide (Sigma, St. Louis, MO, USA) both used as references in the experiment where ampicillin was used as an antibacterial drug, which is known to inhibit prokaryotes organisms while cycloheximide was used as an antifungal drug, which is known to inhibit eukaryotic organisms. The MIC measurement was determined for compounds with inhibition zones >12 mm using a two-fold serial dilution technique [40]. The inhibition zone diameters values cited in Table 4 are attributed to the tested original concentration (1 mg/mL) as a preliminary test and the MIC (μg/mL) values are recorded in Table 5.

### MIC measurement

The microdilution susceptibility test in Müllere-Hinton Broth (Difco) and Sabouraud dextrose broth (Difco) at pH 7.4 was used for the determination of the antibacterial and antifungal activities, respectively [38,39,41]. Stock solutions of the tested compounds, ampicillin and cycloheximide, were prepared in DMSO at a concentration of 1000 μg/mL. Each stock solution was diluted to prepare serial two-fold dilutions at concentrations in the range of 500–3.125 μg/mL. The microorganism suspensions at approximately 10^5^ CFU/mL concentrations were inoculated to the corresponding 96-well plates. The sealed microplates were incubated at 37°C for 24 h for antibacterial activity and at 25°C for 48 h for antifungal activity in a humid chamber. At the end of the incubation period, the MIC values were recorded as the lowest concentrations of the substance that will inhibit the visible growth of the microorganisms (had no visible turbidity). Control experiments with DMSO and uninoculated media were run parallel to the tested compounds under the same conditions.

## Conclusions

In this study, a general rule for the synthesis of 2-amino-5-arylazo-6-aryl substituted nicotinic acids and pyridazinones was established using 3-oxo-2-arylhydrazonopropanals as precursors. Moreover, a novel route to pyrido[2,3-*d*]pyrimidine was achieved. The antimicrobial activities of the resulting novel 2-amino-5-arylazonicotinates, pyridazinone, and pyrido[2,3-*d*]pyrimidine derivatives were investigated with the hope of discovering new structure leads that could serve as antibacterial and antifungal agents. The results of the antimicrobial activities revealed that most of these compounds were found to exhibit strong inhibitory effects on the growth of the Gram-positive bacteria especially *B. subtilis*. Compounds **1a, 8a-g**, **10a-c**, **15b**, and **16** showed considerable antimicrobial activity against *B. subtilis* (Gram-positive bacteria). The results of biological evaluations demonstrated that most of these compounds had promising antimicrobial activities against Gram-positive bacteria.

## Competing interests

The authors declare that they have no competing interests.

## Authors’ contributions

The current study is an outcome of the constructive discussion between all authors. HMI carried out the synthesis, purification, and characterization of the compounds by MS, ^1^H NMR, ^13^C NMR spectral analyses, and the X-ray single crystal analysis. All the authors read and approved the final manuscript.

## Supplementary Material

Additional files 1 CIF file of compound 2a. Click here for file

Additional files 2 CIF file of compound 2h. Click here for file

Additional files 3 CIF file of compound 8a. Click here for file

Additional files 4 CIF file of compound 8b. Click here for file

Additional files 5 CIF file of compound 8c. Click here for file

Additional files 6 CIF file of compound 8h. Click here for file

Additional files 7 CIF file of compound 10a. Click here for file

Additional files 8 CIF file of compound 11a. Click here for file

Additional files 9 CIF file of compound 12. Click here for file
